# Dysregulation of WTI (−KTS) is Associated with the Kidney-Specific Effects of the *LMX1B* R246Q Mutation

**DOI:** 10.1038/srep39933

**Published:** 2017-01-06

**Authors:** Gentzon Hall, Brandon Lane, Megan Chryst-Ladd, Guanghong Wu, Jen-Jar Lin, XueJun Qin, Elizabeth R. Hauser, Rasheed Gbadegesin

**Affiliations:** 1 Duke Molecular Physiology Institute, Durham, NC, United States; 2 Division of Nephrology, Duke University Medical Center, Durham, NC, United States; 3 Department of Pediatrics, Duke University Medical Center, Durham, NC, United States; 4 Department of Pediatrics, Wake Forest Baptist Medical Center, Winston Salem, NC, United States.

## Abstract

Mutations in the *LIM homeobox transcription factor 1*-*beta (LMX1B*) are a cause of nail patellar syndrome, a condition characterized by skeletal changes, glaucoma and focal segmental glomerulosclerosis. Recently, a missense mutation (R246Q) in *LMX1B* was reported as a cause of glomerular pathologies without extra-renal manifestations, otherwise known as nail patella-like renal disease (NPLRD). We have identified two additional NPLRD families with the R246Q mutation, though the mechanisms by which *LMX1B*_*R246Q*_ causes a renal-specific phenotype is unknown. In this study, using human podocyte cell lines overexpressing either *myc*-*LMX1B*_*WT*_ or *myc*-*LMX1B*_*R246Q*_, we observed dominant negative and haploinsufficiency effects of the mutation on the expression of podocyte genes such as *NPHS1, GLEPP1*, and *WT1*. Specifically, we observed a novel *LMX1B*_*R246Q*_-mediated downregulation of *WT1*(−*KTS*) isoforms in podocytes. In conclusion, we have shown that the renal-specific phenotype associated with the *LMX1B*_*R246Q*_ mutation may be due to a dominant negative effect on *WT1*(−*KTS*) isoforms that may cause a disruption of the *WT1* (−*KTS*):(+*KTS*) isoform ratio and a decrease in the expression of podocyte genes. Full delineation of the *LMX1B* gene regulon is needed to define its role in maintenance of glomerular filtration barrier integrity.

Steroid resistant nephrotic syndrome (SRNS) is a condition characterized by defects in the glomerular filtration barrier (GFB) which leads to proteinuria, hypoalbuminemia, edema and an inability to achieve clinical remission following standard corticosteroid therapy. SRNS is associated with a number of renal pathologic changes, the most common being focal segmental glomerulosclerosis (FSGS). Globally, SRNS is a major cause of end stage kidney disease (ESKD) requiring dialysis and kidney transplantation. The pathogenesis of SRNS is still being unraveled, however evidence from studies of the familial form of the disease points to defects in glomerular podocytes as central to disease pathogenesis. Familial forms of SRNS are genetically heterogeneous and are associated with mutations in over 25 genes^1^. One of the genes previously associated with familial SRNS is LIM homeobox transcription factor 1-beta (*LMX1B*)^2^,^3^,^4^,^5^.

*LMX1B* is a transcription factor in the LIM-homeodomain family of proteins. The LIM-homeodomain proteins are vital for the normal development of dorsal limb structures, components of the GFB, and the anterior segment of the eyes. Mutations in *LMX1B* are a cause of nail patellar syndrome (NPS) (OMIM 161200), a condition that is characterized by skeletal dysplastic changes, glaucoma and FSGS with type III collagen fibrils in the glomerular basement membrane^2^. It has previously been observed that patients with NPS caused by a *LMX1B* mutation located in the homeodomain showed significantly more frequent and more severe renal involvement compared with patients with mutations outside of this domain^3^. More recently, a renal specific missense mutation (R246Q) in the homeobox domain of *LMX1B (LMX1B*_*R246Q*_) has been reported by different groups in France, United Kingdom and Japan^6^,^7^,^8^,^9^. In each of these families, all of the affected individuals presented with glomerular pathologies without skeletal or other extra-renal manifestations. However, the full spectrum of renal phenotypes associated with the *LMX1B*_*R246Q*_ mutation is unknown and the reason why the mutation causes kidney-limited disease has not been fully elucidated. As part of our effort to fully characterize genes that are important for maintaining the functional integrity of the GFB, we carried out genome-wide linkage studies (GWLS) and whole-exome sequencing (WES) in families with familial SRNS. We identified two families with the renal-limited *LMX1B*_*R246Q*_ mutations with widely variable renal phenotypes. We uncovered patterns of expression that suggest differential effects of the mutation on a panel of podocyte-expressed genes by comparing immortalized human podocyte control cells to immortalized lines stably overexpressing either wild-type *LMX1B (LMX1B*_*WT*_) or *LMX1B*_*R246Q*_. We observed reduced transcription of *WT1, TRPC6, NPHS1*, and *GLEPP1* in *LMX1B*_*R246Q*_-expressing cells in a pattern suggesting a dominant negative effect by the R246Q mutation on *WT1* gene expression and a haploinsufficiency effect on *TRPC6, NPHS1*, and *GLEPP1* gene expression. Additionally, we discovered a novel LMX1B_R246Q_-mediated downregulation of *WT1*(−*KTS*) isoforms in podocytes which may result in an imbalance of the *WT1(−KTS*) isoforms and subsequently this renal-limited phenotype. This study expands our knowledge of the phenotypes associated with the *LMX1B*_*R246Q*_ mutation and provides further insight into the effects of the mutation on the transcriptional regulation of key podocyte genes.

## Results

### Families and linkage analysis

**Figure 1:**
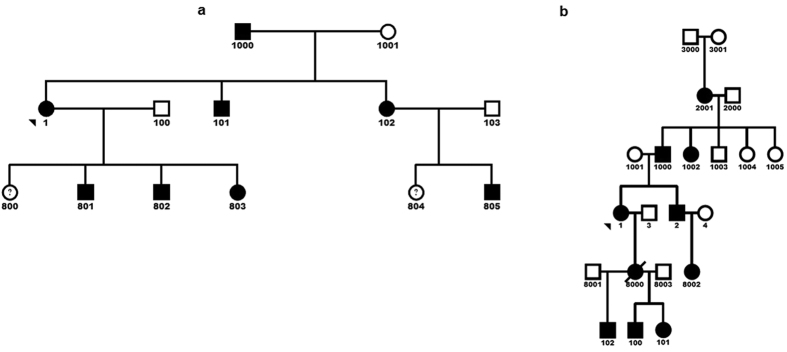
Pedigree of two families with FSGS. **(a** and **b)** There are at least 18 affected individuals in the two families. There is clear male-to-male transmission in both families consistent with an autosomal dominant mode of transmission. Arrowheads identify the proband in both families. DNA samples from individuals 1, 805 and 1000 in Fig. 2A were subjected to whole exome sequencing.

We identified six families with familial SRNS with segregation patterns consistent with autosomal dominant (AD) inheritance. The majority of the affected individuals were diagnosed with FSGS on renal biopsy, and others with findings such as membranous nephropathy, mesangial proliferative glomerulonephritis and minimal change disease. We performed genome wide linkage studies (GWLS) on the six families, the analysis did not yield conclusive evidence of linkage (logarithm of the odds (LOD) score >3) for any individual family. Two families (DUK35705 and DUK34319) however had suggestive logarithm of the odds (LOD) scores of 1.7 and 1.4 on chromosome 9q respectively (Supplementary Figure 1a,b). The pedigrees of these two families are shown in Fig. 1a,b. To identify the smallest candidate region, we performed combined GWLS analysis on the two families and identify a minimal candidate region on chromosome 9q region that spans a physical distance of 58.4 Mb between rs966097 and rs3811133 (Supplementary Figure 2a).

### Phenotypes of the two families with linkage to Chromosome 9q locus

A summary of clinical findings in affected individuals is shown in Table 1. Most of the affected individuals presented with early onset kidney disease with a median age of 12 (range: 3–22) years. Three of the eight affected individuals in Family DUK35705 presented with early onset FSGS while kidney biopsy in the affected individuals in Family DUK34319 showed a wide variety of morphological changes including C1q nephropathy, immune complex glomerulonephritis, and focal global glomerulosclerosis among others (Table 1). In addition to renal diseases, two individuals in DUK35705 have a history of glaucoma and autism spectrum disorder (Table 1). Five out of the thirteen affected individuals in the two families developed ESKD before the age of 25 years. None of the affected individuals who received kidney transplantation had recurrence of the primary kidney disease after transplantation (Table 1).

### Whole exome sequencing (WES) and targeted sequencing

The DNA from three affected individuals in Family DUK35705 were subjected to whole-exome sequencing (WES) using the Illumina TruSeq platform. After excluding variants with minor allele frequencies >1% in the dbGaP database, 68 shared variants remained between the three individuals, of which one variant is the missense variant *c.737G* > *A p.R246Q* in LIM homeobox transcription factor 1-β (*LMX1B*) gene (Supplementary Figure 2b). The complete list of the 68 shared variants is provided in Supplementary Table 1 and the filtering algorithm in Supplementary Table 2. *LMX1B* gene mutations are the cause of nail patellar syndrome (OMIM 161200). Direct sequencing of all affected members of Families DUK35705 and DUK34319 showed that the variant segregated with the disease in both families. We sequenced exons 4 to 6 of *LMX1B* that encode for the DNA binding domain of LMX1B protein in 130 additional families with non-syndromic FSGS, we did not find additional families with mutations in *LMX1B,* thus mutations in exons 4 to 6 of *LMX1B* are responsible for 1–2% of AD FSGS in our cohort. We did not find any other segregating variants in the chromosome 9q region (Supplementary Figure 2a).

### Modifier genes

Since it has been suggested that variants in other genes may be responsible for the variability in renal phenotype associated with *LMX1B* mutations^5^, we searched for presumed pathogenic rare variants (MAF < 1%) in all known FSGS genes, *COL4A3, COL4A4 and COL4A5* in the exomes of three individuals in 35705. We found a rare variant *G545A* in the *COL4A4* gene in one individual in Family DUK35705. Direct sequencing of all other affected individuals in this family showed that only one individual carried this variant. This individual was reported as having membranous nephropathy on light microscopy. There was no obvious difference in the phenotype of this individual and the other members of the family, therefore it appears that modifier genes play a limited (if any) role in these families.

### Phenotypes associated with kidney specific *LMX1B* mutations

We conducted an extensive literature search to identify studies describing the renal limited phenotype associated with *LMX1B* mutations^6^,^7^,^8^,^9^. We identified four studies reporting three mutations in the *LMX1B* DNA binding homeodomains, these studies reported on six families with *LMX1B* mutations. Four out of the six families had the *R246Q* mutation while the other two families carried *R246P* and *R249Q* mutations. These four studies reported a diversity of renal biopsy findings including minimal change disease (MCD), FSGS, mesangial proliferative glomerulonephritis, C1q nephropathy among others (Table 2).

### R246Q mutation and the expression of key glomerular filtration barrier (GFB) genes

**Figure 2:**
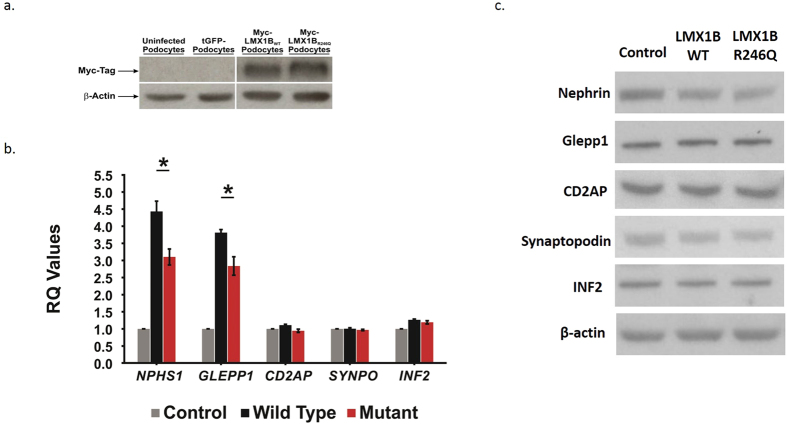
Effect of R246Q mutation on podocyte related gene transcription. (**a**) Western blot confirming expression of the viral transgene with the presence of the MYC tag in *myc*-*LMX1B*_*WT*_ or *myc*-*LMX1B*_*R246Q*_ infected immortalized human podocyte cells compared to uninfected and lentiviral control podocytes (top). β-actin was used as a loading control for all samples (bottom). Blots were cropped to exclude lanes that are not relevant to this paper, the uncropped blot is displayed in Supplementary Figure 6. **(b)** Graph depicting average Relative Quantity (RQ) values for *NPHS1, GLEPP 1, INF2*, and *SYNPO* in *myc*-*LMX1B*_*WT*_ or *myc*-*LMX1B*_*R246Q*_ cells (N ≥ 3). Error bars = SEM *p ≤ 0.05, **p ≤ 0.001 using t-test. **(c)** Immunoblotting showing reduced translation of nephrin (n = 7, p = 0.027) but not GLEPP1 (n = 4, p > 0.05), CD2AP (n = 3, p > 0.05), synaptopodin (n = 3, p > 0.05) and INF2 (n = 3, p > 0.05) in *myc*-*LMX1B*_*R246Q*_ podocytes compared to *myc*-*LMX1B*_*WT*_expressing cells. β-actin was used as a loading control for all samples and unedited blots are shown in Supplementary Figure 6.

To characterize the functional effects of the *LMX1B R246Q* mutation, we examined the changes in LMX1B transcriptional regulation induced by the *R246Q* mutation. To accomplish this, we generated conditionally immortalized human podocyte cell lines stably overexpressing either *myc*-*LMX1B*_*WT*_ or *myc*-*LMX1B*_*R246Q*_ via lentiviral infection. Total RNA was collected from both *LMX1B*-overexpressing lines and from differentiated controls (i.e. uninfected podocytes and tGFP-expressing podocytes). The infection was verified by immunoblot analysis for myc-tag expression and quantification of *LMX1B* mRNA levels in LMX1B-overexpressing podocytes. The levels of *LMX1B* overexpression were similar between the *myc*-*LMX1B*_*WT*_ and *myc*-*LMX1B*_*R246Q*_ infected cells (Fig. 2a and Supplementary Figure 3). We did not detect any obvious differences in the morphology of the F-actin cytoskeleton in the *myc*-*LMX1B*_*WT*_ and *myc*-*LMX1B*_*R246Q*_podocytes by immunofluorescence examination. In addition, subcellular localization of Nephrin, CD2AP, GLEPP1 and WT1 were similar between the two cell lines (Supplementary Figure 4).

We evaluated for alterations in the transcription of several key podocyte genes in the *myc*-*LMX1B*_*WT*_ and *myc*-*LMX1B*_*R246Q*_-overexpressing podocytes by quantitative Real Time PCR (qPCR). *NPHS1, TRPC6*, and *GLEPP1* gene expression was upregulated in both *myc*-*LMX1B*_*WT*_ and *myc*-*LMX1B*_*R246Q*_-expressing podocytes. However, the expression of *NPHS1, TRPC6* and *GLEPP1* was significantly reduced in *myc*-*LMX1B*_*R246Q*_-expressing podocytes compared to *myc*-*LMX1B*_*WT*_-expressing podocytes, suggesting that the mutation may exert a haploinsufficiency effect on the transcriptional regulation of these genes. (Fig. 2b and Supplementary Figure 5). Conversely, no significant changes in gene expression were observed in *INF2, CD2AP, SYNPO* or *COL4A4* between *myc*-*LMX1B*_*WT*_ and *myc*-*LMX1B*_*R246Q*_-overeexpressing podocytes. (Fig. 2b and Supplementary Figure 5). The reduction in *NPHS1* expression and lack of change in *CD2AP, SYNPO* and *CD2AP* was confirmed at the protein level by immunoblot analysis (Fig. 2c). There was no difference in expression of GLEPP1 between the wild type and the mutant cell line (Fig. 2c).

### R246Q mutation and the expression of *WT1* gene

**Figure 3:**
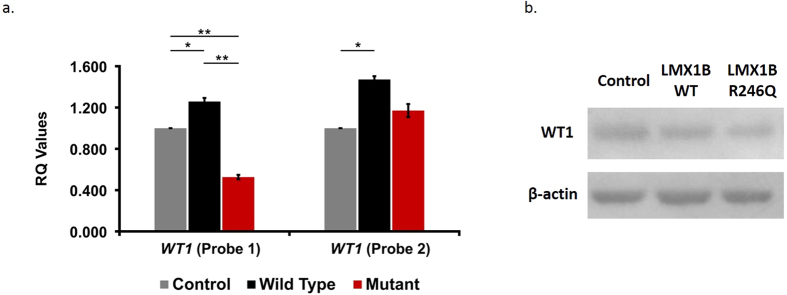
Dominant negative WT1 isoform specific reduction due to R246Q mutation in *LMX1B*. (**a)** The specific reduction of WT1(−KTS) isoform can be seen in this graph depicting average (RQ) values for WT1 (+KTS and −KTS) (WT1 Probe 1) and WT1 (+KTS only) (WT1 Probe 2) expression in *myc*-*LMX1B*_*WT*_ or *myc*-*LMX1B*_*R246Q*_ podocyte cells compared to control podocyte cells (uninfected and lentiviral control) (N ≥ 3). Error bars = SEM, *p ≤ 0.05, **p ≤ 0.001 using t-test. (**b)** The reduction in expression of WT1 protein in *myc*-*LMX1B*_*R246Q*_cells compared to *myc*-*LMX1B*_*WT*_ cells is shown by immunoblotting (n = 6, p = 0.038). β-actin was used as a loading control for all samples and unedited blots are shown in Supplementary Figure 6.

A different pattern of gene expression was uncovered for the regulation of the Wilms’ Tumor 1 gene (*WT1*). In *myc*-*LMX1B*_*WT*_-expressing podocytes, *WT1* was modestly increased compared to the control cell line (RQ of 1.26 vs 1.00, p = 0.0032) (WT1 probe 1 Fig. 3). Conversely, in myc-LMX1B_*R246Q*_-expressing podocytes, *WT1* was significantly decreased relative to wild-type (RQ of 0.53 vs 1.26, p = 0.0002) and controls (RQ of 1.00 vs 0.53, p < 0.00001) (WT1 probe 1 Fig. 3) suggesting a dominant negative effect of the mutation on the regulation of *WT1* gene expression. This reduction of *WT1* expression in myc-LMX1B_*R246Q*_-expressing podocytes compared to *myc*-*LMX1B*_*WT*_-expressing podocytes was confirmed at the protein level by immunoblot analysis (Fig. 3).

**Figure 4:**
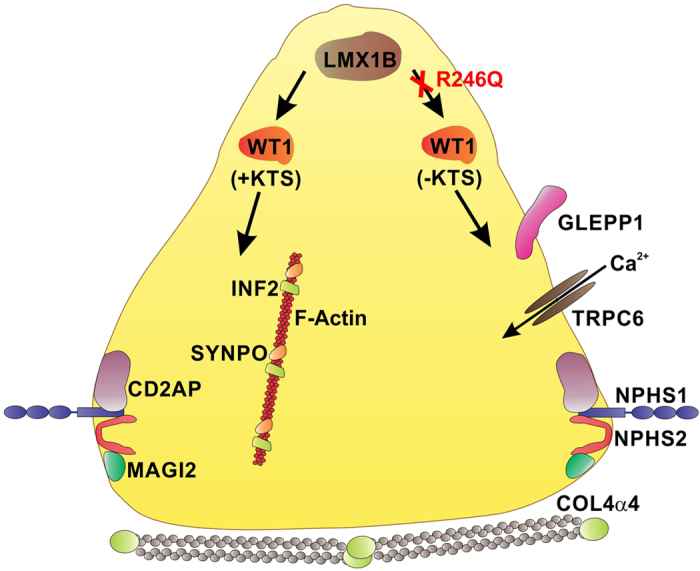
Schematic representation of the proposed mechanism for development of NPLRD due to the *LMX1B*_*R246Q*_mutation. The R246Q mutation causes a specific reduction of the WT1(−KTS) isoform in podocytes leading to a down regulation of key podocyte genes such as *NPHS1, GLEPP1*, and *TRPC6*. This reduction of podocyte gene expression may also be amplified by a reduction in other transcription factors and reduced transcriptional activity of the *LMX1B* transcription factor itself. Through distinct or redundant mechanisms the presence of WT1(+ KTS) isoforms along with potential contributions from *LMX1B* and other transcription factors is able to maintain expression of some podocyte genes such as *CD2AP, INF2, SYNPO* and *MAGI2*.

In a previous study, we identified *synaptopodin (SYNPO*) as a target gene of WT1D, a +KTS isoform of WT1^10^. Despite the apparent dominant negative effect of *LMX1B*_*R246Q*_ on *WT1* gene expression, there was no effect on *SYNPO* mRNA expression. This finding suggests that *LMX1B* may selectively modulate *WT1*-mediated gene transcription via the isoform-specific regulation of *WT1* expression in podocytes. To test this hypothesis, we performed qPCR using an assay designed to detect only the +KTS isoforms (WT1 probe 2 Fig. 3). The expression of +KTS isoforms was increased in *myc*-*LMX1B*_*WT*_and *myc*-*LMX1B*_*R246Q*_ expressing podocytes relative to controls and did not show the dramatic *LMX1B*_*R246Q*_ induced reduction that was observed in the initial *WT1* screen. (WT1 probe 1 Fig. 3). Taken together, these findings suggest that *LMX1B* regulates *WT1* expression in an isoform-specific manner to selectively influence the expression profile of key podocyte genes (Fig. 4).

## Discussion

Advances in genome science and the availability of powerful next-generation sequencing tools for genomic research have accelerated the pace of gene discovery in hereditary nephrotic syndromes. To date more than 25 single genes have been linked to the development of SRNS^1^. Their discovery not only improves our understanding of disease mechanisms, but also provides data allowing better classification and treatment of diseases^11^. Mutations in *LMX1B* was previously identified as a cause of NPS. More recently, missense mutations in the homeodomain of *LMX1B* have been shown to cause a kidney-limited phenotype without extra renal manifestations. So far, twenty-seven cases from six families have been reported with kidney-limited phenotypes due to mutations in exons of *LMX1B* that encode for the homeodomain of the protein. In the present study, we report two additional families with 18 affected individuals. The predominant *LMX1B* mutation in the present report and the six families in previous reports is the *R246Q* mutation (6 of 8 families). Families with this mutation are predominantly of European descent, suggesting that this may be a founder mutation in this population. In our cohort of patients with non-syndromic AD SRNS, mutations in the *LMX1B* homeodomain are responsible for up to 2% of cases, similar to the report of Boyer *et al*.^6^.

The phenotypes associated with mutations of the homeodomain have been described using varied terminology including the term nail patella-like renal disease (NPLRD)^8^. NPLRD is associated with classic transmission electron microscopy findings of diffuse irregular thickening of the glomerular basement membrane with patchy electron lucent areas and irregular deposition of type III collagen fibrils. However, because light microscopy is a more widely available modality for examining renal biopsy especially in resource poor countries, it is important to report the spectrum of light microscopy findings associated with these mutations. In this study different light microscopic findings, including FSGS, MCD, membranous nephropathy and immune complex glomerulonephritis, were described in our patients. Reasons for this observation are not known but it may be due to factors such as the timing of kidney biopsy and/or the presence of modifier genes affecting phenotypes. In line with this speculation, we searched for rare variants in key podocyte and GBM genes that have been associated with SRNS. We identified a single rare variant in *COL4A4 (G545A*) in only one individual with no significant phenotypic variability from other affected family members without the variant. Further studies in a larger cohort of patients are needed to further elucidate the role of modifier genes.

The mechanisms by which mutations in the homeodomain region of *LMX1B* induce renal specific phenotypes have not been fully investigated. Using luciferase assay, Isojima *et al*. suggested that the mutation resulted in *LMX1B* haploinsufficiency^8^. However, a comprehensive survey of the *LMX1B* gene regulon in podocytes has not been reported. In this study we generated human podocyte cell lines stably expressing *myc*-*LMX1B*_*WT*_ and *myc*-*LMX1B*_*R246Q*_ to evaluate the functional effects of the R246Q mutation on *LMX1B*-mediated transcription on collagen alpha 4 and a panel of key podocyte genes. We showed a reduced expression of *NPHS1, TRPC6,* and *GLEPP1* in immortalized podocyte cell line expressing the *myc*-*LMX1B*_*R246Q*_ compared to the *myc*-*LMX1B*_*WT*_, suggesting that the mutation may exert a haploinsufficiency effect on the expression of these genes. We were able to confirm this LMX1BR246Q induced reduction in *NPHS1* and *WT1* expression at the protein level by immunoblot however, there was no change observed in GLEPP1 protein expression. The reason for the disparity in GLEPP1 expression is unclear, but may be due to a prolonged half-life of the GLEPP1 protein in podocytes. Conversely, we did not identify any significant differences in RNA or protein expression between the myc-*LMX1B*_*WT*_ and *myc*-*LMX1B*_*R246Q*_ overexpressing cell lines in other podocyte genes such as *CD2AP, SYNPO*, and *INF2*. Our expression findings are consistent with the observations by Isojima *et al*.^8^. Notably, the pattern of *WT1* gene regulation is different from all the other genes examined. Specifically, the *LMX1B*_R246Q_ mutation exerts a haploinsufficiency effect on *TRPC6, NPHS1*, and *GLEPP1* expression while for *WT1*(−*KTS) isoforms,* the LMX1B_R246Q_ mutation exerts a clear dominant negative effect.

The alternate splicing of leucine, threonine and serine (KTS) amino acids in a critical area between the third and fourth zinc finger domains of WT1 is a highly evolutionarily conserved splicing event^12^,^13^,^14^. However, this archetypal program of translational processing may be insufficient to explain the transcriptional effects of the LMX1B_R246Q_ mutation on *WT1* expression. A possible explanation for LMX1B-mediated isoform-specific *WT1* gene regulation is the use of alternative promoters. These cis-regulatory elements facilitate diversification of gene expression through influences on the efficiency of transcription initiation, pre-mRNA processing, transcript composition, and mRNA turnover^15^. Additionally, these promoter elements have been shown to influence tissue-specific gene expression and developmental gene activity^16^,^17^. It is estimated that 53% of human genes contain alternative promoters and Pal *et al*. have demonstrated that alternative transcription exceeds alternative splicing in generating transcriptome diversity in the developing and adult cerebellum^18^,^19^. Although intragenic alternative promoters have been identified within the *WT1* gene, the role of *LMX1B* in the activation of these regulatory elements is unknown^12^.

Previous studies have shown distinct functional roles and transcriptional targets for WT1(+KTS) and WT1(−KTS) isoforms^20^,^21^,^22^,^23^,^24^. The WT1(−KTS) isoforms have been shown to act as traditional regulators of transcription for genes such as *NPHS1, NPHS2*, and *SYNPO*^23^,^25^,^26^,^27^. Alternatively, the WT1(+KTS) isoforms display reduced DNA binding potential and have been shown to be required for transcription of other podocyte genes such as *SYNPO, MAGI2* and *SCRIB* and may also participate in RNA processing^10^,^21^,^22^,^23^,^24^,^25^,^26^,^27^. It is clear that the ratio of WT1(−KTS) and WT1(+KTS) isoforms is crucial for proper kidney development and function, as a decrease in the +KTS isoform is believed to be responsible for the development of FSGS in Frasier syndrome^28^,^29^. Frasier syndrome is characterized by male pseudohermaphroditism and progressive glomerulopathy due to a mutation in intron 9 of the *WT1* gene that results in reduced WT1 (+KTS) splicing events. In mouse models of KTS isoform specific knockdown, elimination of either isoform leads to a severe disruption of glomerular development and podocyte differentiation^23^,^24^. These findings suggest that an altered WT1 isoform ratio may be one of the mechanisms by which the *LMX1B*_*R246Q*_ mutation produces a kidney-specific effect in NPLRD patients (Fig. 4). Additional studies will be required to further examine the potential alterations in KTS isoform ratio and to unravel which of these podocyte gene expression changes are due to direct alterations in *LMX1B* transactivation potential versus alterations in other transcriptional regulators such as *WT1*.

Contrary to previous reports, we found some individuals with extra renal pathology such as autism and glaucoma in one of the two families. Glaucoma is one of the manifestation of classical NPS, but we are not aware of the report of an association of autism with *LMX1B* mutation in NPS. An association between autism and common variants in *LMX1B* has been reported in a small cohort of patients^30^. It is therefore likely that the finding of autism in our patients may represent effects of other variants in *LMX1B* and other modifier genes. Alternatively, it is possible that the renal-specific mutations may dysregulate the transcription of genes that are important during the development of the podocytes and basement membranes in the kidney, eyes and brain.

In conclusion, we reported two additional families with renal specific mutations in *LMX1B* and showed wide variability in renal pathologic findings within family and between families. Based on these series of experiments, we proposed that the constellation of different glomerular changes in a family with hereditary kidney disease may be a pointer to a diagnosis of kidney specific *LMX1B* mutation. We provide evidence that the renal specific phenotype associated with this mutation may be due to dominant negative effect on *WT1*(−KTS) specific isoforms causing a disruption of the WT1 isoform ratio and subsequent decrease in key podocyte related genes. These findings suggest that a comprehensive delineation of the LMX1B regulon in podocytes may uncover novel transcriptional regulatory patterns and improve our understanding of podocyte LMX1B transcriptional activity in health and disease.

## Materials and Methods

### Institutional review board approval and participants enrollment

Institutional Review Board approval was obtained from the Duke University Medical Center (Durham, NC, USA) and informed consent was obtained from all subjects. All methods were performed in accordance with the relevant guidelines and regulations. Inclusion criteria and determination of affection status are as previously reported^10^,^31^,^32^.

### Linkage analysis

A genome-wide linkage scan was performed using the informative markers in the Illumina HumanOmniExpress-24 genotyping beadchip assay (Illumina Inc., San Diego, California). The list of the informative markers is available on request. Genotyping was performed on 60 individuals from six families comprising of 36 affected individuals and 24 unaffected individuals (unaffected and unknown status). Two-point and multipoint LOD scores were calculated for all the informative SNP markers. A rare dominant model was assumed and a conservative “affecteds-only” analysis was performed.

### Whole-exome sequencing

Whole exome sequencing was performed on three affected individuals in family Duke 35705 using standard protocol. We used the Agilent All Exon 50MB kit and sequenced to at least 75x coverage using one lane of a HiSeq 2000 sequencer. Reads were aligned to the Human Reference genome (HG 18) using the BWA software^33^. Single nucleotide variants were called usingSAMtools^34^. The variants were annotated to Ensembl 50_36 l using SequenceVariantAnalyzer (SVA) and were analyzed using the ATAV software^33^.

### Sanger sequencing

Potential disease causing variants identified by whole-exome sequencing were confirmed by Sanger sequencing. All sequences were analyzed with the Sequencher software (Gene Codes Corp Ann Arbor, MI).

### Conditionally Immortalized Human Podocyte Culture

Conditionally immortalized human podocytes were cultured as described previously^10^.

### mRNA Extraction and Quantitative Real Time PCR

Total RNA was manually extracted from differentiated podocyte cells using an RNeasy Mini kit (Qiagen, Valencia, CA). Subsequently, 0.5 *μ*g of total RNA was reverse transcribed into cDNA utilizing the RT system (Promega Corporation, Madison, WI) with oligo(dT) primers, according to the manufacturer’s protocol. Quantification of mRNA by real-time PCR was performed using the ABI VIAA7 system (Applied Biosystems, Foster City, CA). PCR reactions were performed in a final volume of 10 *μ*l, consisting of 2 *μ*l cDNA, 2.5 *μ*l RNAse and DNAse free water, 0.5 *μ*l of 20× TaqMan Gene Expression Assays, and 5 *μ*l of TaqMan 2× PCR Master Mix (both Applied Biosystems). Probe sequences are listed in Supplementary Table 3. Each individual experiment was performed at least three times, in triplicate or quadruplicate. Relative expression of the target genes was analyzed by normalizing to the housekeeping gene *β*-*Actin*.

### Lentiviral Constructs and Infection

Standard molecular cloning methods were used to replace the ubiquitin-EGFP of FUGW 19 with CMV-turboGFP, CMF-myc-hLMX1B_WT_ and CMV-LMX1B_R246Q_. Lentivirus was made and conditionally immortalized human podocytes were transduced as described previously^32^. Infected and uninfected controls were treated with 2 ug/ml puromycin to select for cells expressing the transgene construct.

### Immunoblotting

Conditionally immortalized podocyte were differentiated per established protocols (PMID: 19955187), harvested, and treated with lysis buffer (Pierce Biotechnology, Rockford, IL) supplemented with phosphatase/protease inhibitor cocktail, 1:100 dilution (Cell Signaling Technology) and 1:100 PMSF (Sigma-Aldrich) for 15 min on ice. Cells were then spun at 14,000 RPM for 10 mins at 4 °C (Eppendorf). Protein Immunoblotting was performed as described previously using a MYC tag antibody 1:000 (Cell Signaling Technology), rabbit polyclonal nephrin antibody1:1000 (Abcam), mouse monoclonal Glepp1 antibody 1:1000 (Santa Cruz), mouse monoclonal WT1 antibody 1:300 (Santa Cruz), rabbit polyclonal CD2AP 1:1000 (Abcam), rabbit polyclonal Synaptopodin antibody 1:800 (Santa Cruz), rabbit polyclonal INF2 antibody 1:1000 (Bethyl Laboratories), and mouse monoclonal β-actin antibody 1:3000 (Sigma-Aldrich)^10^.

### Immunofluorescence

Conditionally immortalized human podocytes were differentiated per established protocols on collagen I–coated coverslips (BD Biosciences) and processed as described previously^10^. Cells were incubated with mouse monoclonal WT1 antibody (Santa Cruz), mouse monoclonal nephrin antibody (Santa Cruz), or polyclonal rabbit CD2AP (Abcam) overnight at 4 °C. Cells were then washed with ice-cold PBS and secondary Alexa Flora 488 antibody was applied (Invitrogen) at a concentration of 1:1000 for 1 hour at room temperature. Cells were then washed four times with PBS at room temperature before addition of phalloidin alexa-568 (Invitrogen) for one hour at RT followed by two PBS washes and then 4′,6-diamidino-2-phenylindole stain at a concentration of 1:20,000 diluted in PBS. Immunofluorescence imaging was performed using a Carl Zeiss AxioImager and the ZenBlue Bioimaging Software.

### Statistical Analyses

All data are represented as the mean ± SEM. Group differences were assessed by the *t* test with unequal variances. Statistical significance was established at *P* < 0.05.

## Additional Information

**How to cite this article**: Hall, G. *et al*. Dysregulation of WTI (−KTS) is Associated with the Kidney-Specific Effects of the *LMX1B* R246Q Mutation. *Sci. Rep.* **7**, 39933; doi: 10.1038/srep39933 (2017).

**Publisher's note:** Springer Nature remains neutral with regard to jurisdictional claims in published maps and institutional affiliations.

## Supplementary Material

Supplementary Data

## Figures and Tables

**Table 1:** Phenotype associated with *LMX1B* mutation in two families with FSGS.

Study ID	Gender	Age onset (yrs)	Proteinuria Y/N	Renal biopsy	Extra renal manifestations	Age at ESKD (yrs)	Transplant Y/N Recurrence Y/N
35705: 1	F	<18	Yes	FSGS	No	18	Y/N
35705: 101	M	<22	Yes	UNK	No	22	Y/N
35705: 102	F	<16	Yes	UNK	Glaucoma	16	Y/N
35705: 801	M	5	Yes	FSGS	Autism	NA	N/NA
35705: 802	M	3	Yes	FSGS	Autism	NA	N/NA
35705: 803	F	6	Yes	NA	No	NA	N/NA
35705: 805	M	<14	Yes	NA	No	NA	N/NA
35705: 1000	M	18	Yes	Memb	Glaucoma	58	Y/N
34319: 1	F	12	UNK	Familial nephropathy	No	22	N/NA
34319: 100	M	4	Yes	FGGS	No	NA	N/NA
34319: 101	F	5	Yes	UNK	No		N/NA
34319: 102	M	2	Yes	Immune complex GN	No	5	Y/N
34319: 8002	F	10	Yes	C1q nephropathy	No	NA	N/NA

F: Female, M: Male, UNK: Unknown, NA: Not applicable, Memb: Membranous nephropathy, FSGS: Focal segmental glomerulosclerosis, FGGS: Focal global glomerulosclerosis, GN: Glomerulonephritis, Y: Yes, N: No, YRS: Years.

**Table 2:** Renal biopsy findings in 45 individuals from eight families with renal limited *LMX1B* mutations.

Renal biopsy findings	N (%)	Mutations	References
FSGS/FGGS	14 (31.1)	R246Q	6, 9, and present study
None specific (CKD)	4 (8.9)	R249Q	6 and 7
MCD	2 (4.4)	R246P	6
Nail patellar like renal disease	1 (2.2)	R246Q	8
Mesangial proliferative GN/C1q nephropathy	2 (4.4)	R246Q, R249Q	7, and present study
Membranous GN	1(2.2)	R246Q	Present study
Immune complex GN	1 (2.2)	R246Q	Present study
Familial nephropathy	1 (2.2)	R246Q	Present study
Unknown	19 (42.2)	R246Q, R249Q	6, 7, and, present study

GN: Glomerulonephritis, MCD: Minimal change disease.
