# Supplementary material for: Dysregulation of WTI (−KTS) is Associated with the Kidney-Specific Effects of the LMX1B R246Q Mutation
Source: Sci Rep. 2017 Jan 6;7:39933. doi: 10.1038/srep39933 (PMC5216339; doi:10.1038/srep39933)
Supplement: Supplementary Data [file srep39933-s1.pdf]

DYSREGULATION OF *WT1* (-KTS) IS ASSOCIATED WITH THE KIDNEY-SPECIFIC EFFECTS OF THE *LMX1B* R246Q MUTATION

Gentzon Hall[1,2#,] Brandon Lane[1,3#], Megan Chryst-Ladd[1,3], Guanghong Wu[1,3], Jen-Jar Lin[4], XueJun Qin[1], Elizabeth R. Hauser[1], Rasheed Gbadegesin[1,3*]

[1]Duke Molecular Physiology Institute,

[2]Division of Nephrology, Duke University Medical Center, Durham, NC

[3]Department of Pediatrics, Duke University Medical Center, Durham, NC

[4]Department of Pediatrics, Wake Forest Baptist Medical Center, Winston Salem, NC

[#]These authors contributed equally to this manuscript

*To whom correspondence should be addressed:

rasheed.gbadegesin@duke.edu

Supplementary material

Supplementary Figure 1: Genome wide linkage studies showing suggestive locus on chromosome 9q (dark arrow) in **(a)** DUK35705 and in **(b)** DUK34319.

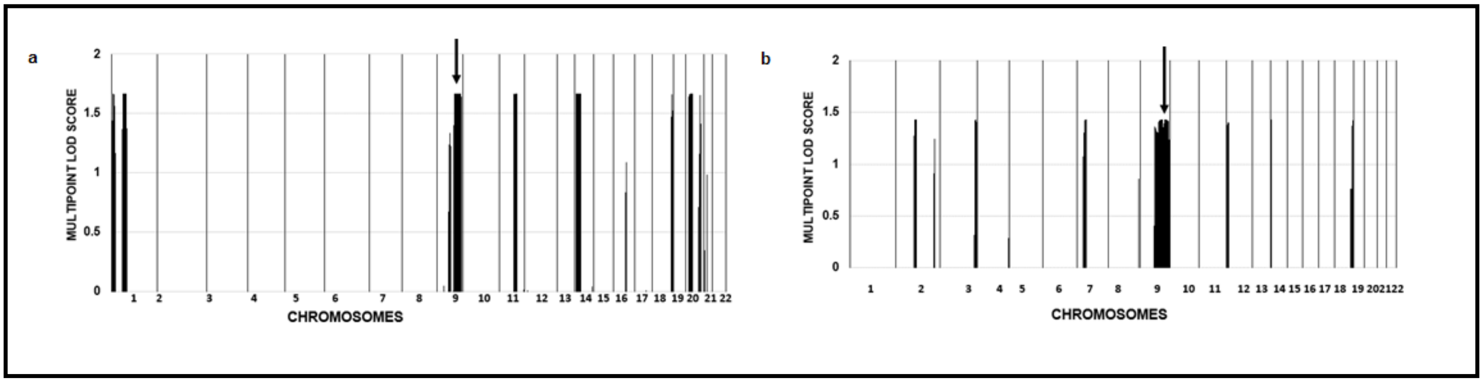

Supplementary Figure 2: Renal specific R246Q LMX1B mutations in two families with FSGS. (a) Genome-wide linkage studies in two families with SRNS yielded a minimal candidate region on chromosome 9q (black arrow). Chromosome numbers are shown on the X axis and LOD scores are on the Y axis. *LMX1B* is located in the chromosome 9q peak. **(b)** Segregating missense heterozygous mutation *c.737G>A p.R246Q is* found in *LMX1B* gene in families 35705 and 34319. Mutant sequence on the left and wild-type sequence on the right.

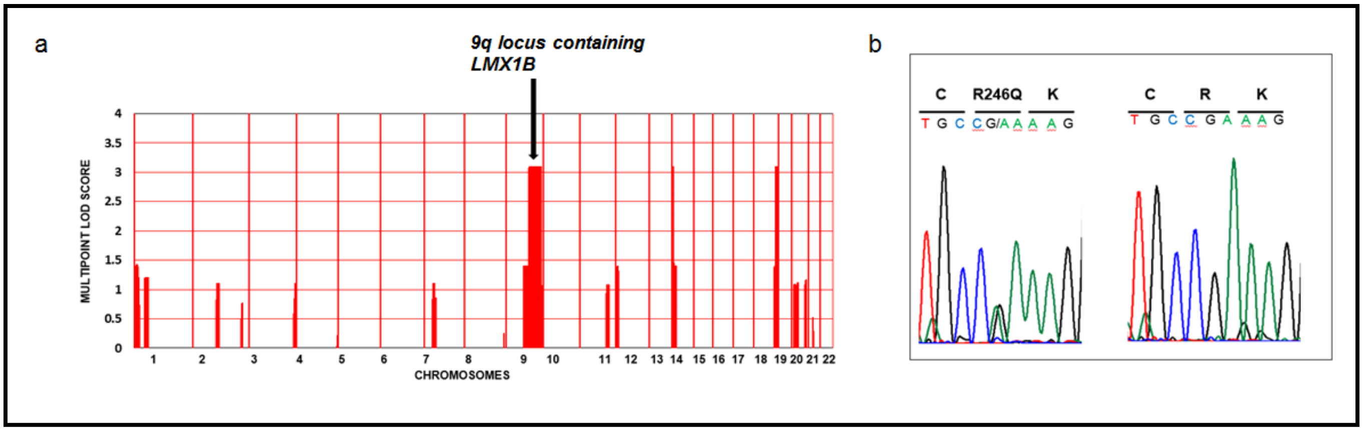

Supplementary Figure 3. RQ values for LMX1B expression in control, *myc-LMX1B*$_{WT}$, and *myc-LMX1B*$_{R246Q}$ podocyte cells. Expression was quantified relative to control cells which have an RQ value of 1.

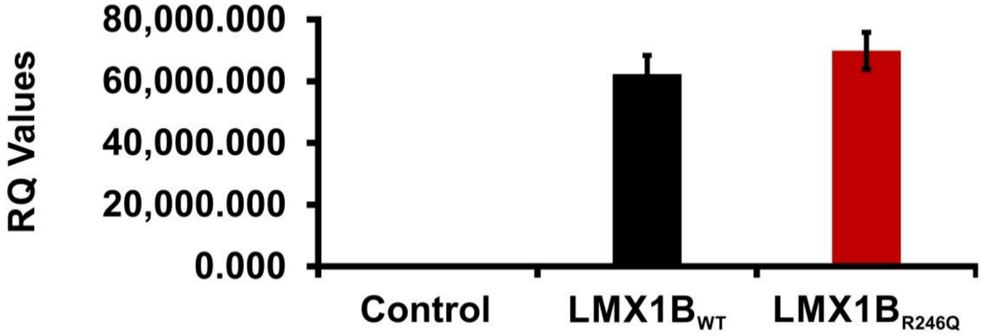

Supplementary Figure 4: Immunofluorescence staining of *myc-LMX1B*$_{WT}$ and *myc-LMX1B*$_{R246Q}$ cell lines for F-actin showed structurally normal F-actin cytoskeleton in both cell lines and similar subcellular localization of nephrin, CD2AP, and WT1.

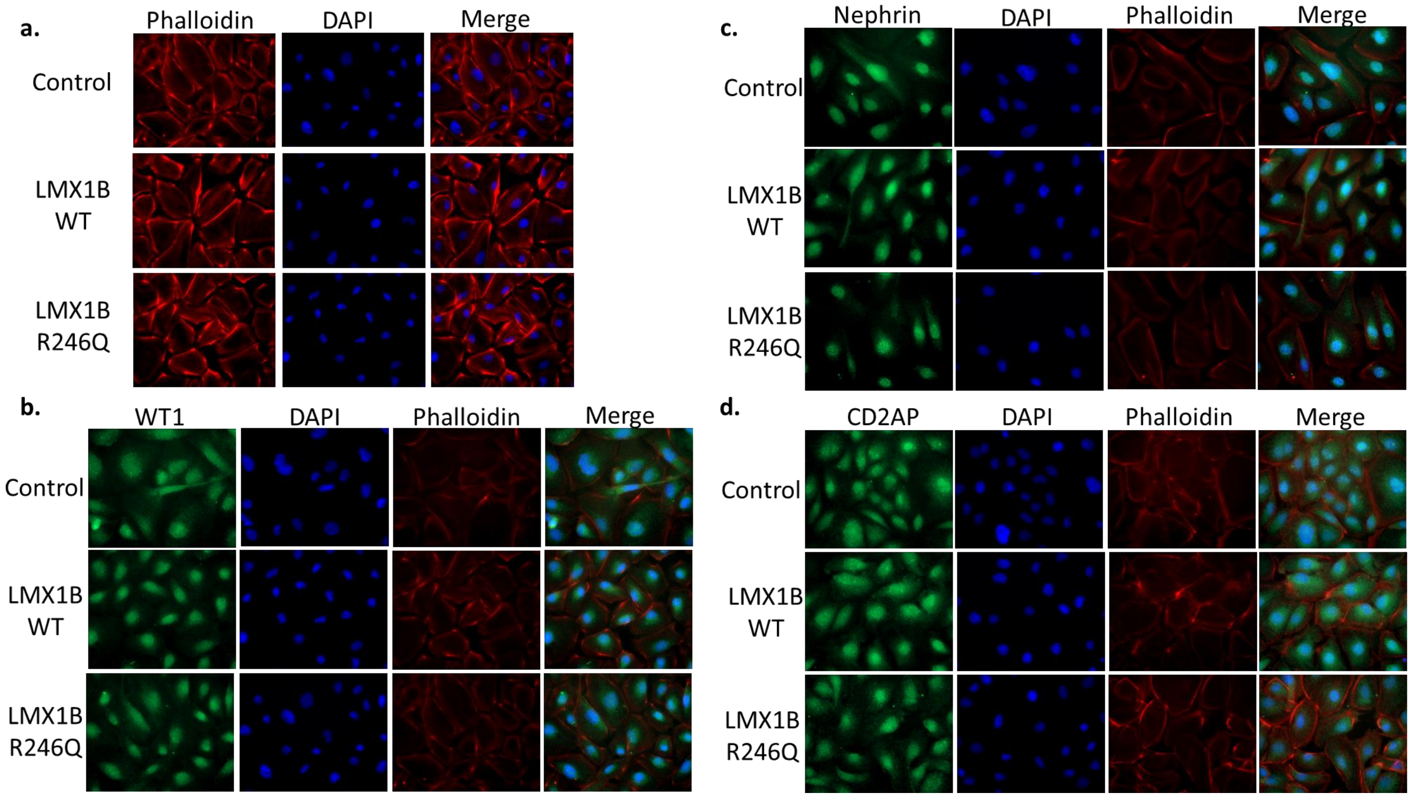

Supplementary Figure 5: Expression of *COL4A4* and *TRPC6, in myc-LMX1B$_{WT}$* and *myc-LMX1B$_{R246Q}$*-overexpressing podocytes (n=3, *p value <0.05).

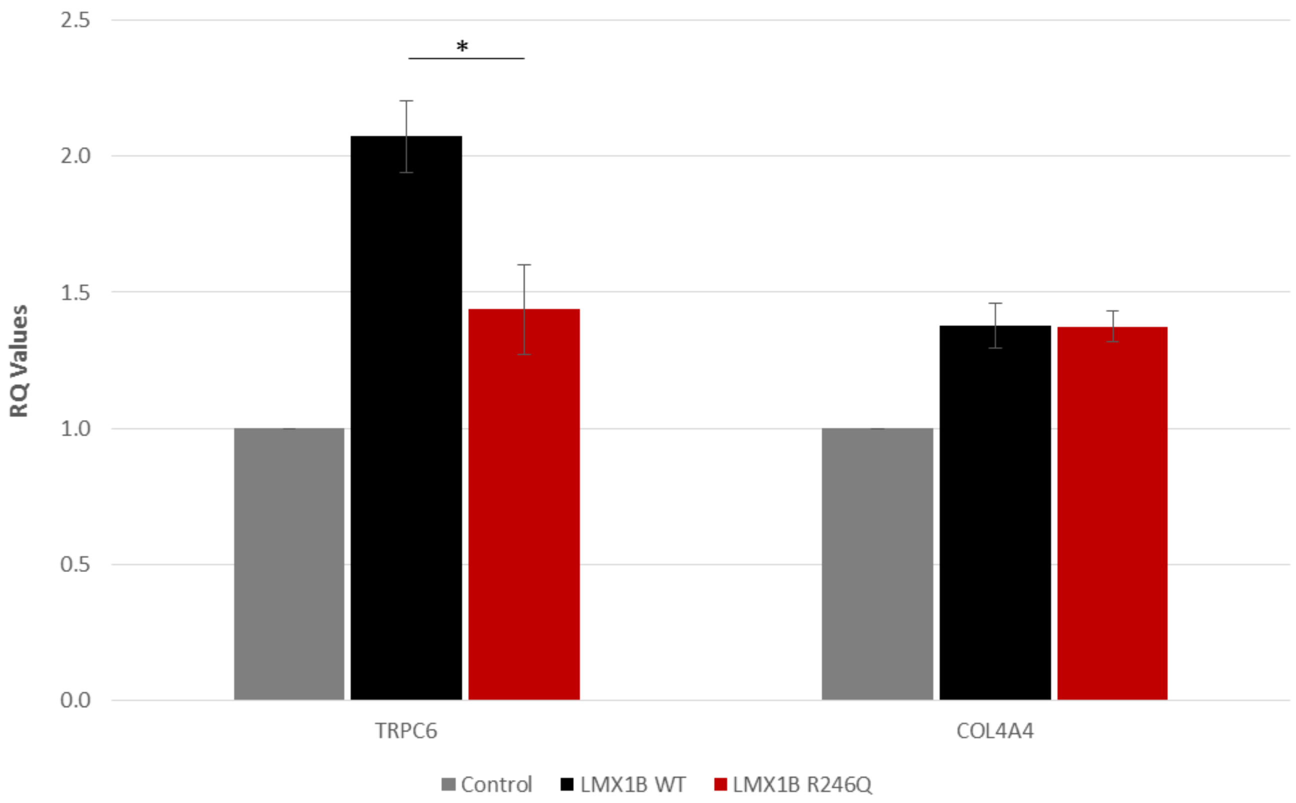

Supplementary Figure 6. Unedited blots shown in the paper. a. Figure 2a, b. Figure 2b, c. Figure 3

a.

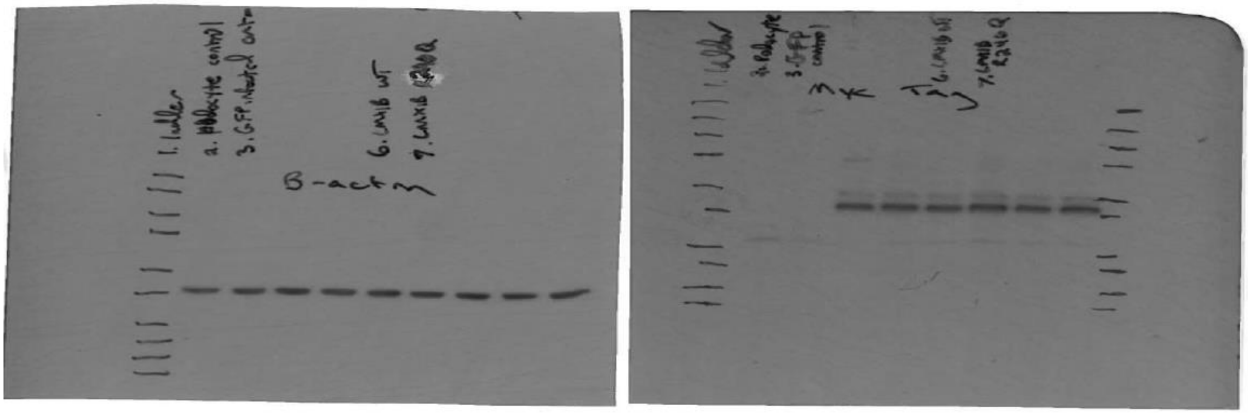

b.

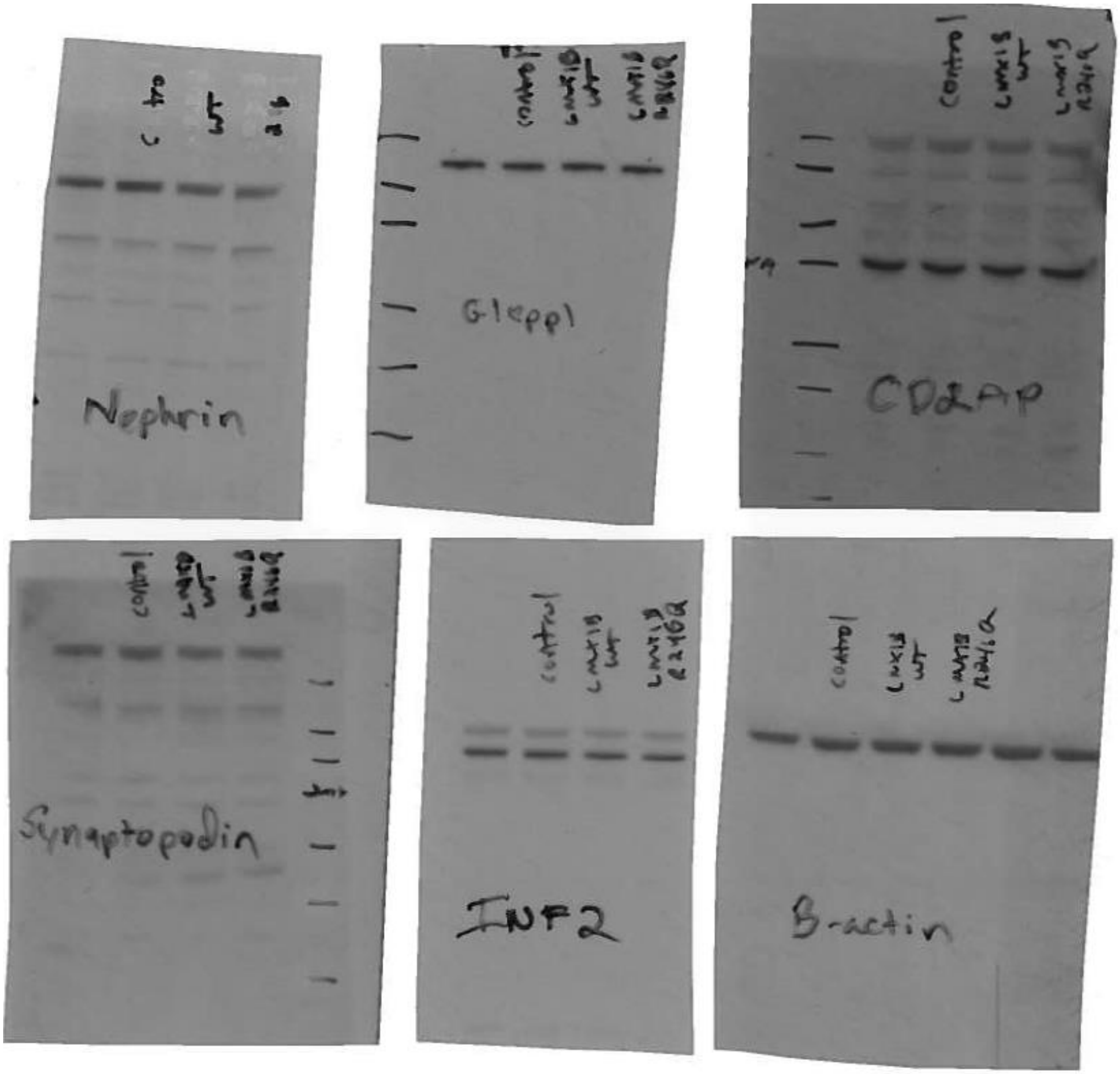

c.

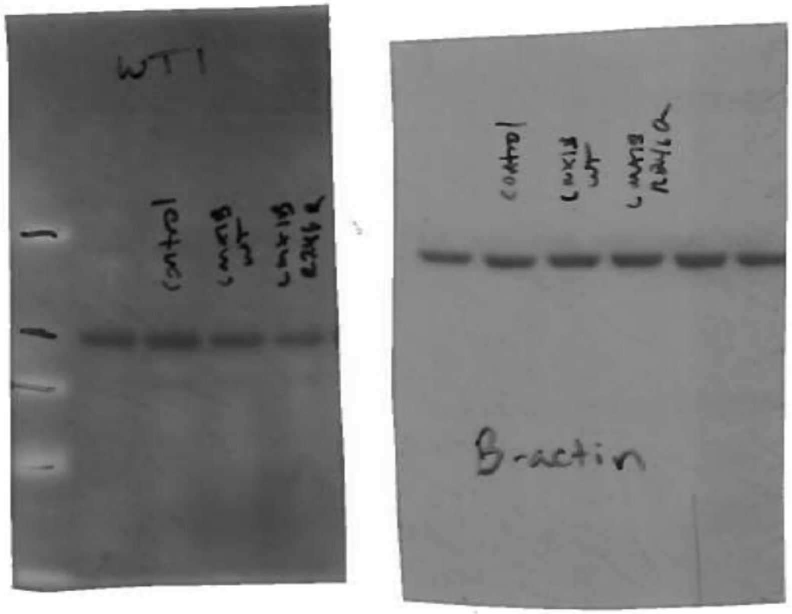

Supplementary Table 1: 68 rare variants shared by three affected individuals in Family 35705

	VARIANT ID	RS ID	REF ALLELE	GENOTYPE	ExAC ALL MAF	GENE	FUNCTION
1	20_45865096_T	rs371544404	C	het	2.47194E-05	ZMYND8	NON SYNONYMOUS
2	20_29624070_A	rs113131305	G	het	0.000207727	FRG1B	NON SYNONYMOUS
3	10_99342400_T	rs149812913	C	het	0.001540997	ANKRD2	NON SYNONYMOUS
4	3_188592145_A	rs9830664	G	het	0.001998712	LPP	NON SYNONYMOUS
5	13_77531179_G	rs116855637	A	het	0.003626004	IRG1	NON SYNONYMOUS
6	1_117156459_T	rs61786651	C	het	NA	IGSF3	NON SYNONYMOUS
7	3_75714807_A	rs199780042	G	het	NA	FRG2C	NON SYNONYMOUS
8	20_46275917_C	rs149561356	A	het	0.002652389	NCOA3	NON SYNONYMOUS
9	22_42457056_T	rs121434529	C	het	0.002517265	NAGA	NON SYNONYMOUS
10	20_31652478_A	rs142043656	G	het	0.00560953	BPIFB3	NON SYNONYMOUS
11	9_69423887_G	rs199906260	A	het	NA	ANKRD20A4	NON SYNONYMOUS
12	9_129455598_A	NA	G	het	NA	LMX1B	NON SYNONYMOUS
13	10_117884984_A	NA	G	het	NA	GFRA1	NON SYNONYMOUS
14	16_70884524_G	rs75694658	C	het	NA	HYDIN	NON SYNONYMOUS
15	16_70894087_C	rs149857179	T	het	NA	HYDIN	NON SYNONYMOUS
16	16_70902559_T	rs77739958	C	het	NA	HYDIN	NON SYNONYMOUS
17	16_70902568_T	rs78562971	C	het	NA	HYDIN	NON SYNONYMOUS
18	21_14982721_G	rs200814491	A	het	NA	POTED	NON SYNONYMOUS
19	22_25425282_G	rs116253946	A	het	NA	KIAA1671	NON SYNONYMOUS
20	1_117156585_A	rs139013364	G	het	8.2702E-06	IGSF3	STOP_GAINED
21	2_242128114_T	rs148609049	C	het	0.005786804	ANO7	STOP_GAINED
22	3_75714805_75714805_DEL_G	rs144577984	TG	het	NA	FRG2C	FRAME_SHIFT
23	3_75788150_75788151_INS_G	rs143871834	A	het	NA	ZNF717	FRAME_SHIFT
24	16_70896015_70896015_DEL_A	rs77276171	GA	het	NA	HYDIN	FRAME_SHIFT
25	19_1526613_T	rs2292455	C	het	NA	PLK5	SPLICE_SITE_REGION
26	1_20879457_T	NA	C	het	NA	FAM43B	START_GAINED
27	3_128182378_A	rs13071640	G	het	NA	DNAJB8	START_GAINED
28	3_184429718_G	rs12011	A	het	NA	MAGEF1	START_GAINED
29	4_103790186_T	rs223332	G	het	NA	CISD2	START_GAINED
30	22_41967870_T	rs6002408	C	het	NA	CSDC2	START_GAINED
31	22_44893563_G	rs5765689	A	het	NA	LDOC1L	START_GAINED
32	1_117142868_T	rs61730489	C	het	NA	IGSF3	STOP_GAINED
33	X_55172687_C	rs1047037	T	het	0	FAM104B	NON SYNONYMOUS
34	1_117142869_G	rs78806598	A	het	2.4888E-05	IGSF3	NON SYNONYMOUS
35	11_18478275_T	NA	G	het	8.23845E-06	LDHAL6A	NON SYNONYMOUS
36	1_117156584_C	rs143106517	T	het	8.27157E-06	IGSF3	NON SYNONYMOUS
37	3_69168656_C	NA	T	het	3.31274E-05	LMOD3	NON SYNONYMOUS
38	3_154055506_G	NA	T	het	6.21305E-05	GPR149	NON SYNONYMOUS

	VARIANT ID	RS ID	REF ALLELE	GENOTYPE	ExAC ALL MAF	GENE	FUNCTION
39	3_172115105_A	rs142343118	G	het	0.000156528	FNDC3B	NON SYNONYMOUS
40	20_54945542_T	rs200181472	C	het	0.00023888	AURKA	NON SYNONYMOUS
41	16_71012855_C	rs149870093	T	het	0.000250108	HYDIN	NON SYNONYMOUS
42	1_117142736_G	rs138851517	A	het	0.000490914	IGSF3	NON SYNONYMOUS
43	1_22055176_C	rs141036007	T	het	0.000930976	USP48	NON SYNONYMOUS
44	16_70917931_G	rs142251979	C	het	0.001054189	HYDIN	NON SYNONYMOUS
45	4_113436014_C	rs146017617	G	het	0.001200828	NEUROG2	NON SYNONYMOUS
46	1_248652071_C	rs199723306	G	het	0.005025282	OR2T5	NON SYNONYMOUS
47	17_1840917_T	rs201050462	C	het	0.001504916	RTN4RL1	NON SYNONYMOUS
48	9_34724905_A	rs200921257	G	het	0.001997337	FAM205A	NON SYNONYMOUS
49	22_17265124_C	rs114306778	A	het	0.003682408	XKR3	NON SYNONYMOUS
50	12_108105893_A	rs11547909	G	het	0.003873477	PWP1	NON SYNONYMOUS
51	1_207110449_T	rs12748810	C	het	0.00517396	PIGR	NON SYNONYMOUS
52	1_17596817_T	rs145296609	C	het	0.00486213	PADI3	NON SYNONYMOUS
53	3_75713564_C	rs143803036	T	het	NA	FRG2C	NON SYNONYMOUS
54	3_75714298_G	rs114375735	A	het	NA	FRG2C	NON SYNONYMOUS
55	3_75714337_G	rs150591814	T	het	NA	FRG2C	NON SYNONYMOUS
56	3_75714702_G	rs150428518	A	het	NA	FRG2C	NON SYNONYMOUS
57	3_75714809_T	rs117327874	C	het	NA	FRG2C	NON SYNONYMOUS
58	3_75714819_A	rs115599848	G	het	NA	FRG2C	NON SYNONYMOUS
59	3_75714825_A	rs115693429	G	het	NA	FRG2C	NON SYNONYMOUS
60	3_75714831_A	rs138192454	G	het	NA	FRG2C	NON SYNONYMOUS
61	3_75714840_G	rs200756071	A	het	NA	FRG2C	NON SYNONYMOUS
62	3_75714843_A	rs201904817	T	het	NA	FRG2C	NON SYNONYMOUS
63	8_17407880_T	NA	A	het	NA	SLC7A2	NON SYNONYMOUS
64	9_69423755_C	rs202189725	G	het	NA	ANKRD20A4	NON SYNONYMOUS
65	9_69423792_T	rs78465713	A	het	NA	ANKRD20A4	NON SYNONYMOUS
66	16_70917855_G	rs138915898	A	het	NA	HYDIN	NON SYNONYMOUS
67	22_17264565_T	rs115201131	G	het	NA	XKR3	NON SYNONYMOUS
68	22_21742684_T	rs200610234	C	het	NA	RIMBP3B	NON SYNONYMOUS

Supplementary Table 2: Filtering algorithm for Whole exome sequencing data

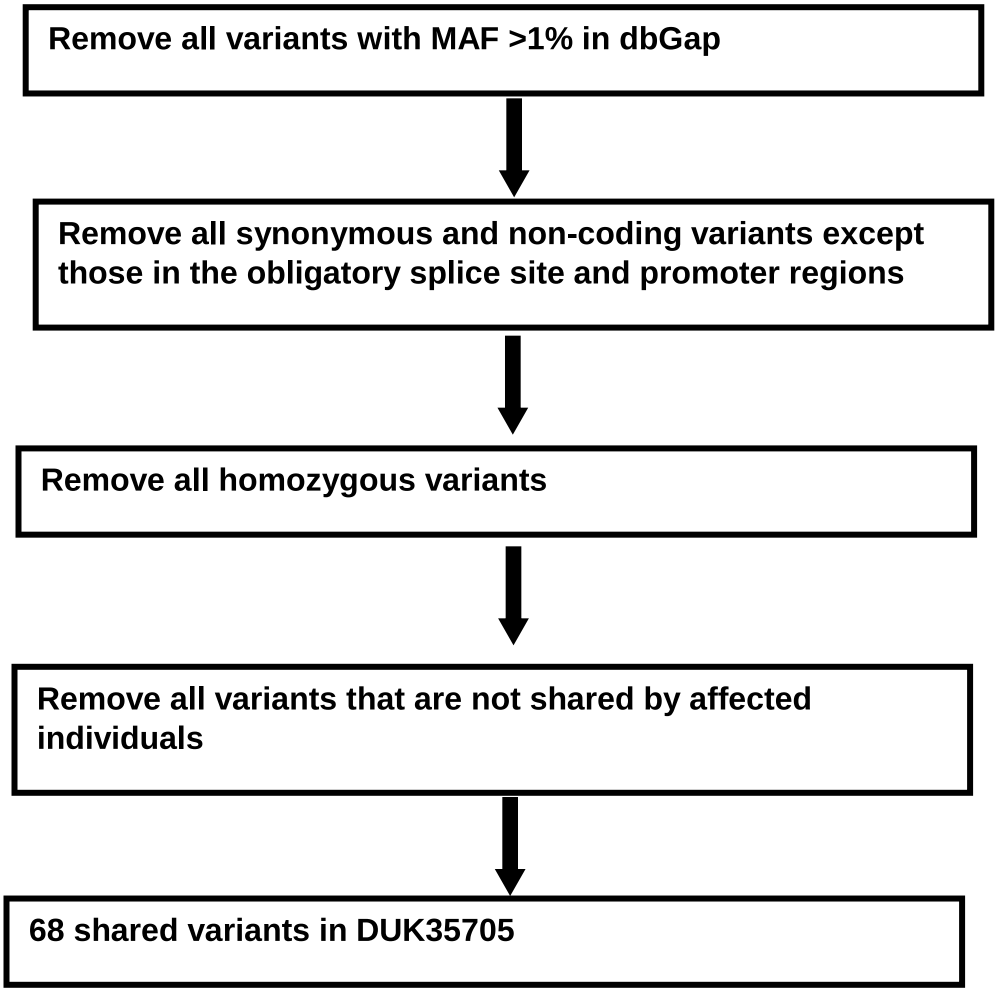

Supplementary Table 3. Sequences and ABI identification numbers for the qPCR probes used in this analysis.

Probe ID	Target	Sequence
Hs00536292_g1	INF2	ggaccactacaagacggtgtgcagccagcagtaccgcttcagcattgtcatgaacgagctctccggcag cgacaacgtgccctacgtggtcaccctgcttag
Hs00961451_m1	CD2AP	tttttattgaaacttgtattatttttaaagagatctatactataaatatggtgatatatttacaagtaatctgt taagatatactatttgagagggacagattagccttttagtaactatagtcactactttttccataatgcat
Hs00702468_s1	SYNPO	ggtggccagtgaggaggaagaggtaccactggtggtttatctaaaggagaatgcagcactgctgacagcc
Hs00387817_m1	NPHS2	ccaaatcctccggcttaggggcctgtgagtggcttcttgtcctcatttccctgctcttcatcatcatgac
Hs00190446_m1	NPHS1	aggtctccaccagccttctggagaacctgaagaccagctgcccacagagccaccttcaggaccctcg
Hs01011885_m1	COL4A4	ctggcagggtcttgccttcccgtatttagcacgctgccctttgcctactgcaacatccaccaggtgtgccactat
Hs01022542_m1	COL4A3	tcacaagtgcaggttctgagggcaccgggcaagcactggcctcccctggctcctgcctggaagaattccg
Hs00958189_m1	PTPRO (GLEPP1)	gtacatttttatccatcagtgtgtgcaactgatgtggatgaagaagaagcagcagttctgcatcagtgatg tcatatacgagaatgttagc
Hs00158750_m1	LMX1B	agctcttcgcggccaagtgcagcggctgcatggagaagatcgcccccaccgagttcgtgatgcgggcgct ggagtgcgtgtaccacctgggctgcttct
Hs00988473_m1	TRPC6	ggcatgatatgggctgaatgtaaagaaatctggactcagggccccaaggaatatttgtttgagttgtggaa catgcttgattttggtatgttagcaattttcgcagcatcattca
Hs01060665_g1	ACTB	ggcgtgatggtgggcatgggtcagaaggattcctatgtgggcgacgaggcccagagcaagaga
Hs01103751_m1	WT1	aatttataccaaatgacatcccagcttgaatgcatgacctggaatcagatgaacttaggagccaccttaaag
Hs01103755_m1	WT1 (+KTS isoforms only)	agcccttcagctgtcggtggccaagttgtcagaaaaagtttgcccggtcagatgaat
